# The Rejection Template of Working Memory Operates after Attention Capture

**DOI:** 10.3390/bs12110436

**Published:** 2022-11-08

**Authors:** Jiachen Lu, Jing Wang, Weidong Li, Jingjing Li

**Affiliations:** 1 School of Psychology, South China Normal University, Guangzhou 510631, China; 2 School of Physical Education, Guangzhou University, Guangzhou 510006, China

**Keywords:** working memory, attention capture, attention suppression, biased competition model, visual attention theory

## Abstract

Although scientists know that information stored in working memory guides visual attention, how this is accomplished is still under debate. Specifically, there is a dispute between the Biased Competition Model and Visual Attention Theory. The current study used two experiments to resolve this controversy based on previous research. Experiment 1 found that although inverse efficiency scores for High and Low numbers of memory distractors were both longer than the Baseline (no memory distractors), they did not significantly differ from each other. This indicated that memory might guide attention via a capture-then-global-inhibition process. Experiment 2 addressed the possibility that the findings resulted from the time needed to reject the interfering objects by requiring both memory-matching and memory-mismatching conditions to be rejected under a highlighted target. This result showed that the memory-matching condition resulted in longer search times than the memory-mismatching condition, indicating an attention-capture effect based on working memory. Together, the two experiments support the idea that when multiple memory-matching distractors in a search array first capture an individual’s attention, it then acts as a template that allows the individual to suppress all interfering items that containing memory information holds. This study supports the Biased Competition Model early on in visual search. However, the late stage of visual search supports the Visual Attention Theory. These advance our knowledge regarding the relationship between working memory content and attention.

## 1. Introduction

In recent years, the interaction between working memory and attention is a topic that has been explored by researchers in cognitive neuroscience and biopsychology. Previous studies have revealed brain areas involved in working memory that overlap with those involved in attention, with both processes using the posterior and frontal regions [1,2,3,4]. This overlap with activated brain regions between working memory and attention to some extent determines their functional interactions. Specifically, most results demonstrate memory-driven attentional capture on the basis of content-specific representations [5,6,7].

Past studies have proposed distinct theoretical explanations regarding the process through which information stored in working memory can influence visual attention [8,9]. The Biased Competition Model states that the working-memory content can serve as an attention template and that the memory-matching stimulus will prioritize capture attention [10,11]. For example, in one classic study, participants were asked to remember a color or shape feature and then identify it. Before the identification task, the memory retention phase, a search task is completed. The results show that, compared with matching novelty features, the search efficiency decreases when the distractors match the memory features. The authors explained that memory-matched distractors preferentially captured attention, resulting in slower search response time, even if memory features were unrelated to the search task [5]. The Visual Attention Theory (TVA) suggests that observers use target templates in working memory to bias perceptual choices by increasing the attention weight of template features to process similar items. In other words, observers can use the content of visual working memory to set the attention weight of a feature in working memory to zero (or a very small value), preventing similar items from receiving the benefit of attention [12,13,14]. Therefore, this paper is concerned with the issue of working memory, whether salient stimuli are either entered into WM (targets), or rejected from WM (distractors), according to a pre-set bias towards targets relative to distractors that operate automatically, or whether the influence of attention persists such that items in WM can be strengthened, or discarded, throughout the duration of WM. The first option is consistent with the biased competition model, and the second is with Bundesen’s TVA model. Woodman and Luck [15] found that when a participant knows that the item that they are keeping in memory will interfere with their search for a target, their memory template can be used as a template for rejection. Further, in Experiment 4 of their study, when the total number of searches was fixed, search reaction time was inversely related to the number of memory-matching distractors. When the total number of stimuli is constant, the more memory distractors there are, the fewer target analogs there are. After rejecting the memory distractors, only a small number of target analogues need to be searched. A similar finding was found in the research of Arita et al. [16].

However, these results in Woodman and Luck [15] can be obtained either by directly suppressing attention to the interfering stimulus in advance or by first being attracted to one of the interfering stimuli and then rejecting all the distractors containing the memory information as a whole. To address the inferential limitation, Carlisle and Woodman [17] used event-related potential to explore the relationship between memory and attention. Additionally, they found that an N2pc to memory-matching items was only observed when attending to the memory-matching items consistent with the observer’s goals. Additionally, working memory representations alone are not sufficient to guide early deployments of visual attention to matching inputs. Although N2pc does respond well to early attention selection [18], the N2pc of memory-matching distractors and goals had been confused in the study of Carlisle and Woodman [19]. Specifically, N2pc was obtained by subtracting the ipsilateral N2 from the contralateral one, the target opposite the memory-matching distractors reduces the N2pc of memory-matching distractors. Donohue et al. [19] used magnetoencephalogram technology to explore how the brain inhibits singleton distractors. In their experiments, the target always appeared on the center line of the screen rather than on the sides, thus eliminating interference with goals. Additionally, the result found that singleton distractors can induce significant N1pc components in the early stages of visual search. The author suggested that distractors are not only processed but they are also given temporal priority, with the brain building a robust representation of the to-be-ignored items.

Therefore, it is still controversial whether the rejection template can completely suppress attention, and also, whether the way in which memory or rejection templates regulate attention in complex displays is consistent with the singleton results of Donohue et al. [19]. To this end, we designed two experiments to explore this problem. Experiment 1 used the dual-task paradigm that incorporates working memory and a visual search to explore whether there is a capture effect in the process of rejecting memory distraction. We made changes based on Woodman and Luck [15], adding trials with no memory items as a baseline condition for comparison. Experiment 2 addressed the possibility that the findings resulted from the time needed to reject the interfering objects by requiring both memory-matching and memory-mismatching conditions to be rejected under a highlighted target.

## 2. Experiment 1

Experiment 1 comprised a dual-task paradigm in which visual search tasks were added during the delay phase of a change-awareness task [20]. In the search array, the experiment has three types of items in the search array: a. Targets (target squares inside non-memory objects); b. Non-memory distractors (distractor squares inside non-memory objects); c. Memory distractors (distractor squares inside memory objects). The baseline condition only includes items in ‘a’ and ‘b’, while the Low and High conditions contain all three types. A single-factor, three-level, within-participants design was adopted in which the search task included the following three conditions: (1) The Baseline condition—three search objects of the same color and shape (non-memory objects), one memory object that differed from the non-memory objects in both color and shape, and a search target (an up or down opening-direction square-frame) that appeared randomly inside one of the non-memory objects; (2) the low quantity-matching condition (‘Low’ condition)—same as a baseline but with two memory-distractors whose color and shape matched those of the memory item; (3) the high quantity-matching condition (‘High’ condition)—same as the Low condition, but with four memory-distractors. The colored shape of the non-target-analog items was consistent with the previous memory items in the memory distraction trials. Furthermore, neither the color feature nor the shape feature of the target-analog items were consistent with the previous memory item.

We had the following three hypotheses: (1) According to the Visual Attention Theory, if participants directly suppress attention to the memory-matching distractors, the difference in time to find the target will not be significant between the three conditions. (2) According to the Biased Competition Model, if participants reject the memory templates one by one, search time will increase with the number of distractors that match the memory information. Thus, search time would be fasted in the Baseline condition, followed by the Low condition, and then the High condition. (3) Combining the above two approaches, a memory-matching interference is captured by attention and then the rejection template is used to reject all distractors. Thus, the number of distractors that match the memory information will not affect the search time. Thus, search times for the High and Low conditions would not differ significantly, but both would be longer than that in the Baseline condition.

### 2.1. Experimental Methods

#### 2.1.1. Participants

An a priori power analysis performed by G*Power (power = 0.80, alpha = 0.05, *η*^2^ = 0.45, referring to Woodman and Luck [15]) estimated the minimum sample size to be 15. To have better power, twenty-five undergraduate and graduate students from Liaoning Normal University participated in Experiment 1 (11 males and 12 females, aged between 18 and 25 years). All participants had a normal or corrected-to-normal vision, and no participant had any mental illness or color vision disorder. Participants provided their informed consent before the experiment. The experiment lasted about one hour, and they were given compensation afterward. The entire study was reviewed and approved by the South China Normal University ethics committee, and all methods were performed in accordance with the relevant guidelines and regulations.

#### 2.1.2. Experimental Apparatus and Materials

The entire experiment was carried out in a comfortable and quiet room. All experimental materials were presented on a 17-inch computer monitor with a white background located 57 cm from the participants. The memory and search objects were colored patterns subtending 1.8° × 1.8° of visual angle. The objects comprised six colors (blue, yellow, red, green, purple, orange) and ten shapes (isosceles triangle, pentagon, hexagon, trapezoid, ellipse, remnant, rhombus, parallelogram, cross shape, funnel shape) that were randomly paired, resulting in 60 possible items. After each trial began, the memory shape and the non-distracting search shape were randomly selected from these 60 possibilities.

**Figure 1:**
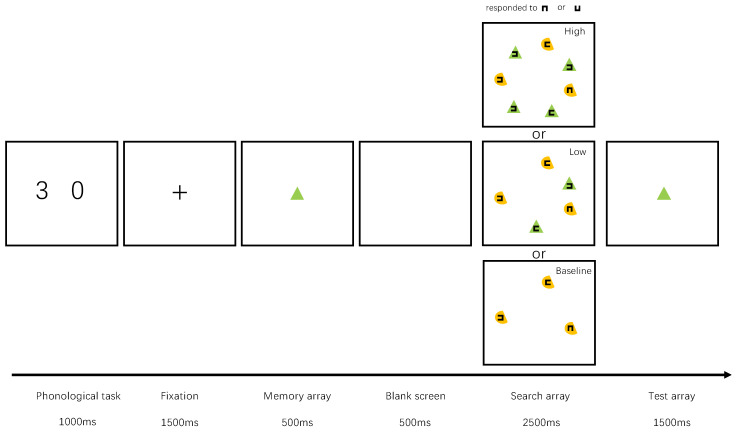
Trial structure of the dual-task paradigm. In this example, there are two kinds of items (non-target items and target items) in the search array. The non-target items share the same color and shape as the memory item and occur only in the memory distraction trials (High or Low) but not in the Baseline trials. The target appeared only inside the target items. The paradigm consisted of three different types of trials.

Items in the search array (as shown in Figure 1) were evenly distributed throughout a virtual disk with an angle of 9°. The distance from each item to the central position was 4.5° and the minimum distance between items was 1.6 to ensure that distractors and targets were well-separated. A square frame with a 0.2° gap was inserted at the 0.6° central position of the colored shapes to serve as the searched-for item. The search array included one target with either an up or down opening-direction square frame and five distractors with either left or right opening-direction square-frames (“5” for up; “1” for down). Two types of items were designed in the search array, namely, non-target-analog items and target-analog items. The target-analog items included a target and two distractors, and they shared the same color and shape. The non-target-analog items included three distractors sharing another color and shapes.

#### 2.1.3. Experimental Procedure

Experiment 1 used a dual-task paradigm that has been commonly used in previous studies [20,21]. The experimental process is shown in Figure 1. First, two different numbers between 0 and 9 (0.8° × 1.2°) were presented on the left and right sides of the monitor (1.8° from the center) for 1000 ms. Participants were required to verbally repeat these two numbers during the rest of the trial to reduce the impact of speech coding on the experimental task [22]. Next, referring to the time setting in the previous study [15], a fixation cross was presented for 1500 ms, followed by the item to be held in memory for 500 ms. Participants were asked to concentrate and memorize the item during its brief appearance. After a subsequent 500 ms blank screen, the search array appears for 2500 ms. The search array consisted of multiple visual stimuli that were randomly distributed at 12 possible points around the edge of a virtual circle with a radius of 3°. Each stimulus was presented 3° from the center and the minimum distance between items was 1.5° to ensure that the items did not interfere with each other. Participants needed to find the target and identify the location of its gap (top or bottom) by pressing the up or down arrow keys on the keyboard (“5” for up; “1” for down). It should be noted that there was only one search target and its shape and color never matched those of the memory item. After the gap location was entered, a 1500 ms memory detection item was presented. Participants pressed the “Z” key if both the color and the shape of the presented item matched that of the memory item and pressed the “X” if it did not (“Z” for memory objects; “X” for non-memory objects). It is important to note that we clearly indicated during the instruction period that the shape and color of the target item would never match the shape and color of the memory item.

The three conditions were presented pseudorandomly for 150 trials such that there were 60 trials for both High and Low conditions and 30 trials for the Baseline condition. Thus, 80% of the trials were experimental trials.

### 2.2. Results

The memory performance accuracies for the three conditions are shown in Table 1. One-way repeated-measures analyses of variance (ANOVAs) showed that there was no significant difference in memory accuracy among the conditions (*F* _(2,48)_ = 0.53, *p* = 0.59, *η*^2^ = 0.02).

**Figure 2:**
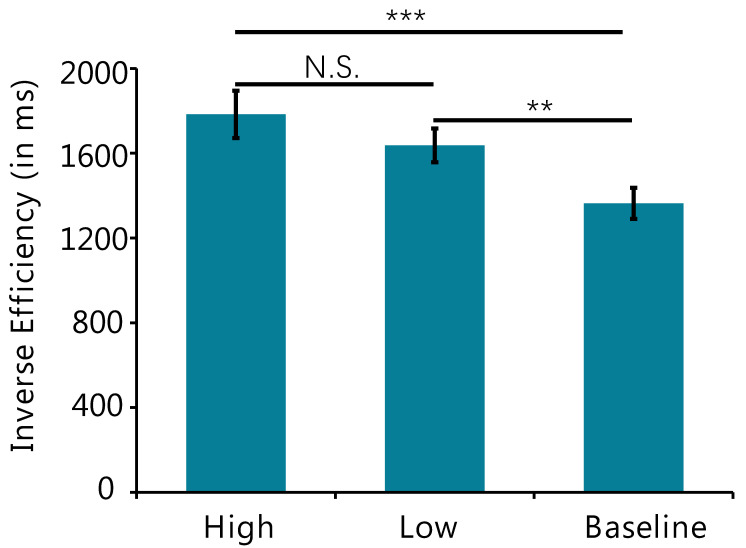
Inverse efficiency (by dividing the mean correct RTs by the proportion of correct responses) across conditions in Experiment 1. *** indicates *p* < 0.001; ** indicates 0.001 < *p* < 0.01; N.S. indicates *p* > 0.1.

Given that we required participants to perform the search task both quickly and accurately, this could involve a trade-off between reaction time and accuracy. We integrated speed and accuracy into a single metric of processing costs, or inverse efficiency score, by dividing the mean correct RTs by the proportion of correct responses [23]. The inverse efficiency scores are shown in Figure 2. One-way repeated-measures ANOVA revealed a significant effect of conditions (*F* _(2,48)_ = 9.47, *p* < 0.001, *η*^2^ = 0.28). Post hoc comparisons by paired-comparison t-test showed that the difference between inverse efficiency scores for the High and the Low condition was not significant (*t* _(24)_ = 1.54, *p* = 0.136, Cohen’s d = 0.63). However, the inverse efficiency scores for both High (*t* _(24)_ = 3.95, *p* < 0.001, Cohen’s d = 1.61) and Low conditions were significantly higher than those for baseline (*t* _(24)_ = 2.97, *p* = 0.007, Cohen’s d = 1.21).

### 2.3. Discussion

Experiment 1 found that when memory content appeared as a distractor in the search task (High and Low conditions), the search efficiency to find the target was slower than when it did not (Baseline). These results indicate that the memory-distractors captured the attention of the participants, which supports the Biased Copetition Model. Previous studies have found that an inhibitory effect occurs only under high probability conditions (80%), whereas a guiding effect occurs under low probability conditions (20%) [21]. In our experiment, the probability of encountering memory-matching distractors was also 80%. Thus, our results were consistent with a suppressive effect of distractors that contain memory information. However, we also found that was not affected by the number of the memory-matching interferents (Low vs. High conditions, i.e., two vs. four interferes). According to the Biased Competition Model, if each memory-distractor is captured by attention, the capture of four should be greater than the capture of two, which would lead to longer scores. However, the experimental results do not support this hypothesis. Rather, our results support that they first capture an individual’s attention, and then act as a template that allows the individual to suppress all interfering items containing memory information.

Woodman and Luck [15] found that the total number of items for different conditions in Experiment 4 of the rejection template was the same, but the total number of items in our experiment was different. Therefore, the total number of items may affect experimental results. However, even if the total number of items differed for high (four) and low (two) numbers of memory-distractors, the time needed to detect the target and respond did not significantly differ. This means that inverse efficiency scores were affected by including distractors that matched the contents of working memory but not by the total number of items.

Additionally, based on the experimental design, the inverse efficiency score findings could have other explanations in that the rejection process might prolong the search time. Because it takes time for the participant to reject memory-distractors, it took longer for the high number of memory-distractors than it did for the low number of memory-distractors. Therefore, Experiment 2 included a contrast condition in which both the memory-matching and the memory-mismatching conditions needed to be rejected and was thus able to explore whether a capture effect exists under the memory-match condition.

## 3. Experiment 2

### 3.1. Experimental Methods

#### 3.1.1. Participants

An a priori power analysis performed by G*Power (power = 0.80, alpha = 0.05, *η*^2^ = 0.28, referring to the result of Experiment 1) estimated the minimum sample size to be 22. Due to the impact of COVID-19, only twenty undergraduate and postgraduate students from Liaoning Normal University participated in Experiment 2 (10 males and 10 females, aged between 18 and 25 years). All participants had the normal or corrected-to-normal vision, and no participant had any mental illness or color vision disorder. Participants provided their informed consent before the experiment. The experiment lasted about one hour, and they were given compensation afterward. The entire study was reviewed and approved by the South China Normal University ethics committee, and all methods were performed in accordance with the relevant guidelines and regulations.

#### 3.1.2. Experimental Apparatus and Materials

**Figure 3:**
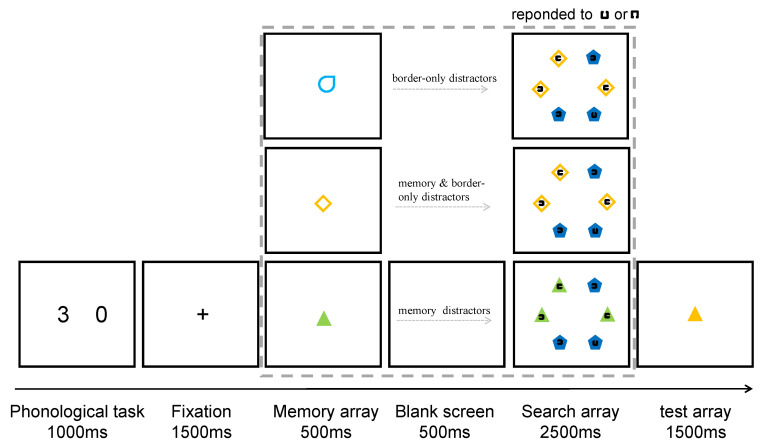
Trial structure of the dual-task paradigm in Experiment 2.

The apparatus and materials of Experiment 2 were similar to those of Experiment 1, except for the following differences. In addition to including the 120 filled-in shapes from Experiment 1, Experiment 2 included border-only versions of these shapes. Thus, there were 240 border-only shape stimuli and 120 filled-in shape stimuli (Figure 3).

#### 3.1.3. Experimental Procedure

The experimental process is shown in Figure 3. The experimental instruments, materials, designs, and procedures were similar to Experiment 1, except for the following changes. The search array in Experiment 2 contains three conditions: (1) Memory Distractors condition, with three identical memory-distractors and three identical non-memory objects, and one of the non-memory objects contained the target; (2) Border-only Distractors condition, with three identical border-only objects and three identical filled-in shape objects, and one of said filled-in shape objects contained the target. This condition did not include any memory distractors; (3) Memory Border-only Distractors condition, which is similar to the Border-only Distractors condition, except for border-only objects will match the memory information.

The three conditions each contain 1 block, 80 trials per block. The order of conditions was random among the participants. It should be noted that we clearly told the participants that the search target would never appear within the border-only objects or the memory objects. There was no reason for the participants to take the initiative to pay attention to either the border-only distractors or the memory distractors. Finally, in order to be consistent with experiment 1, in which the effective rate of reminder is 80%, twenty percent of the trials of one condition will come from the other two conditions. We only analyze the remaining 80% of the trials.

### 3.2. Experimental Results

The memory accuracies for the three conditions are shown in Table 2. Additionally, one-way repeated-measures analyses of variance (ANOVAs) showed no significant difference in memory accuracy (*F* _(2,38)_ = 0.11, *p* = 0.825, *η*^2^ = 0.01).

**Figure 4:**
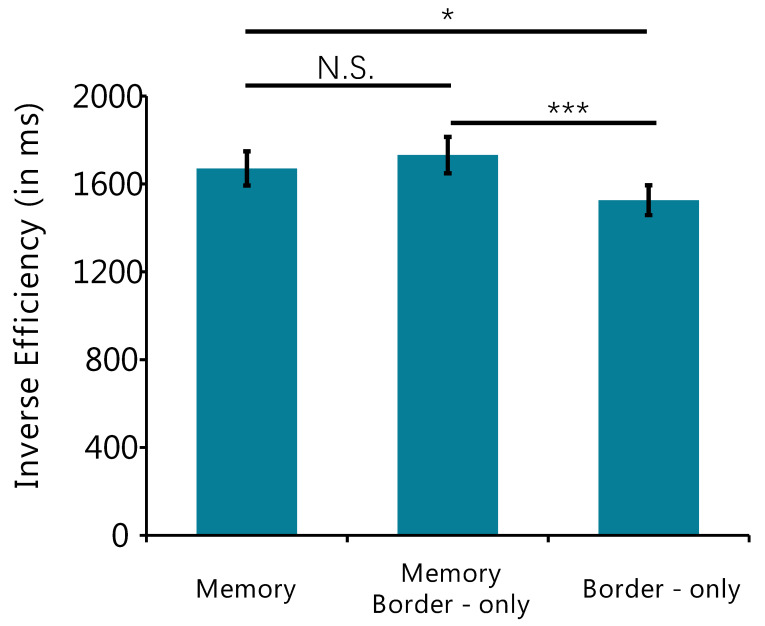
Inverse efficiency for the three conditions. Error bars are SEM. * indicates *p* < 0.05; *** indicates *p* < 0.001; N.S. indicates *p* > 0.1.

The inverse efficiency for the three conditions is shown in Figure 4. Additionally, one-way repeated-measures analyses of variance (ANOVAs) showed a significant difference in inverse efficiency (*F* _(2,38)_ = 6.60, *p* = 0.03, *η*^2^ = 0.26). The paired sample t-test was used for post hoc comparisons analysis, and the results found that the Border-only Distractors condition was significantly less than Memory Distractors condition (*t* _(19)_ = 3.31, *p* < 0.001, Cohen’s d = 1.52) and Memory Border-only Distractors condition (*t* _(19)_ = 2.41, *p* = 0.03, Cohen’s d = 1.11), but the difference between Border-only Distractors condition and Memory Distractors condition is not significant (*t* _(19)_ = 0.13, *p* = 0.90, Cohen’s d = 0.06).

### 3.3. Discussion

Experiment 2 found a lower search efficiency during the Memory Border-only condition than during the Border-only condition. This could mean that when items matching memory content appear again as distractors in a search task, they capture attention, so that the rejection process takes longer. First, the Memory Border-only condition and Border-only conditions both have three identical irrelevant distractors items, which inevitably affects the search efficiency of the participants. Second, the two conditions have the same number of searches, the same presentation positions, and the same number of target items. The only difference is whether they contain distractors that include objects in working memory.

At the same time, the target-analog items are highlighted in these two conditions because the participant is clearly told that the target will not appear in the border-only shape items, and the participant can directly search for filled-in shape items. However, the search efficiency in the Memory Border-only condition is lower than that in the Border-only condition, which confirms the capturing of working memory information. On the other hand, there is no significant difference between the search efficiency in the Memory Border-only condition and in the Memory condition. This suggests that the search efficiency is not affected by the different physical features of the memory distractors. Even if the participant knew that the border-only shape items would not be the target, but rather the filled-in shape items would, it does not affect the capture of border-only shape items with memory information. In Experiments 1, if participants reacted more slowly to the distractor containing the memory information, it could only be said that it took time to reject the distractor, but not that it was because the distractor matched the memory item. In Experiment 2, all conditions required rejecting the same number of distractors. If memory information had no effect on performance, there should be no difference in search efficiency between conditions. However, this is contrary to the experimental results. Therefore, we believe that during the search process, the attention of the participant was captured by the memory-distractors and then released, as attention to the distractors containing the memory information was suppressed.

## 4. General Discussion

This study explored the process of attention-based guidance based on working memory. The two experiments together indicate that this process includes two stages: when a stimulus matching working-memory content appears, it initially captures the viewer’s attention. Subsequently, the viewer suppresses attention to similar stimuli at a later stage. The experiments also indicate that as long as one of the memory-distractors captures their attention, the participant can use the rejection template to reject all similar distractors as a whole. Therefore, we believe that working memory is a guiding process that inhibits the capturing of working overall memory after attention has been captured by it.

### 4.1. Working Memory Acts to First Capture and Then Suppress Attention

Through two experiments we proved that even if there is a rejection effect of working memory contents on attention, there will be a phenomenon of first capturing and then rejecting in this process. In Experiment 1, we found that participants reacted more slowly to the distraction condition with the memory information than to the baseline condition. At the same time, the number of memory distractors (2 or 4) has no significant effect on the search efficiency, indicating that redundant memory distractors are suppressed. That is, only a few memory items capture attention, causing increased memory distractors that do not have a significant impact on the search efficiency. In Experiment 2, we also found that the search efficiency was slower under the matching distractors, which supported that the memory-matching stimuli caused attention capture. Our experiment is supported by previous studies. For example, Lu et al. [21] used an eye-tracking technique and found that the presence of memory distractors would reduce the search time, but the probability of the first fixation point falling on the memory distractors was significantly greater than on target-analog items. In addition, Donohue et al. [19] found that a singleton memory distractor can induce significant N1pc components in the early stages of visual search. The author suggest that distractors are not only processed, but they are given temporal priority, with the brain building a robust representation of the to-be-ignored items. Thus, successful suppression of distractors can only be achieved if distractors are first strongly neural represented.

In addition, our research resolves the dispute on the relationship between the content of working memory and attention in previous studies. In the research of Olivers et al. [5], a memory item was used as a single distractor, and when the memory interferer appears, the search time increases, illustrating the capture of attention by working memory. However, Woodman and Luck [15] found that when the total number of stimuli in the search array remains constant, the greater the number of memory distractors recurring, and the faster the search time. The authors suggest that the participant has an inhibitory effect on the memory distractors. Current research considers that the main reason for the difference is the number of memory distractors in the search array. In order to generate a rejection effect, Woodman and Luck [15] used multiple memory distractors that appeared repeatedly, and the attention is captured by one memory distractor and then rejects the remaining distractors. In turn, the total search time becomes shorter. In the research of Olivers et al. [5], there is only one memory distractor. When the attention is captured by the one, the search response time will be longer. So, the process cannot reflect the inhibitory effect.

Therefore, these two experiments suggest a top-down capture effect of working memory content that occurs before the effective suppression of the memory-matched stimulus. Our study found that early on in visual search, the memory distractor always captures our attention, which supports the Biased Competition Model [24]. However, later on, we can flexibly control our attention, so that the memory distractors became our inhibition template, making us no longer pay attention to relevant information, that is, supporting the visual attention theory. This solves the debate regarding the Biased Competition Model and Visual Attention Theory with respect to inhibition and capture effects.

### 4.2. The Rejection Template and the Overall Rejection Method

Woodman and Luck [15] found that the search time of finding memory distractors (distractor squares inside memory objects) was faster than that of the neutral condition under a high memory burden, which indicates that working memory content can be used as a template for rejection. In Experiment 4 of that study, when the total number of searches was fixed, the more distractors matched with the content of working memory, the faster the search time. These results suggest the existence of rejection templates, but how the rejection template worked during the rejection process was unknown. Specifically, the same results could be obtained by rejecting distractors one by one or by rejecting all of them as a whole.

In contrast to Woodman and Luck [15], the total number of items was not fixed in our current experiment, but the number of items with similar characteristics to the search target was fixed at three. If search time does not depend on the number of memory-distracting items that must be rejected when the number of search-related items (non-memory objects that might include the target) is fixed, this indicates that rejection of distractors is performed as a whole, not one by one. This is indeed what we observed. Regardless of the number of memory-distractors in Experiment 1 (two or four), search time remained relatively constant, indicating that participants rejected all the memory distractors as a whole. We not only extended the findings from Woodman and Luck [15], but also determined that working memory content facilitates rejection of attention after first capturing the memory-related items. Our study expands the field of research and provides new evidence for suppressing the capture of attention based on rejecting existing memory templates.

### 4.3. Supplement to Early Theoretical Models

This study found that working memory content first captured attention and then helped suppress attention. This result supports the Biased Competition Model [10]. According to the Biased Competition Model, contents kept in object working memory will enhance the perceptual representation of matched stimuli through a top-down approach [25]. This process increases the activation of relevant cortical regions in the brain and allows the stimuli that match with the content of working memory to be prioritized, leading to a competitive advantage. In our experiments, the memory item had nothing to do with the search target. However, the memory item still captures the participants’ attention first, then the participant will use the memory item to form a rejection template, which can be used later to reject all instances of the same interfering items. That is, working memory influences attention via a mechanical process, and the working memory content is used as a template for capturing attention. However, when participants know that the target has nothing to do with the memory content, they will suppress the memory target. Thus, there is a flexible control process. Guided search 6.0 of Wolfe [26] provides support for the current results, which suggests that if a target is not found, search terminates when an accumulating quitting signal reaches a threshold. The setting of that threshold is adaptive, allowing feedback about performance to shape subsequent searches. The rejection template reduced the threshold of memory distractors, allowing attention to be focused on the distractor only for short periods of time. Therefore, increasing the number of distractors does not affect the search efficiency.

Unlike the Biased Competition Model, our results are not fully consistent with Visual Attention Theory. The theory holds that individuals can flexibly set the attention weight of features according to the degree of correlation between the memory content and the search targets [11]. When the individual knows that the working memory content is related to the search target, the memory content is set to a very high weight, and the memory content is used as an attention template. When the individual knows that the working memory content is not related to the search target, the memory content is set to a very low weight. The content of working memory serves as a rejection template, thus suppressing attention to the memory-matching stimulus. However, we found that even if the participants knew that the memory content would not be related to the target, it interfered with the search and captured the attention of the participants. This shows that the influence that working memory has on attention is not as flexible and controllable as the Visual Attention Theory predicts. Individuals cannot control their attention by setting different attentional weights according to the correlation between the memory content and the search target.

Finally, our results extend Cognitive Control Theory, which believes that in the early stage of visual search, the content kept in working memory will capture attention [27]. Cognitive control is affected by the difficulty of perceptual processing and the duration of the stimulation interval (the time interval between the memory array and the search arrays) [28]. However, the theory does not explain the way in which memory matching interferes with stimulus detection. With our results, we can add to the theory and say that participants will form a rejection template and adopt overall rejection.

### 4.4. Limitations and Prospective Research

First, we inferred the capture and re-suppression mechanism through which working memory modulates attention by comparing conditions in two experiments. However, search time is a comprehensive result of a variety of cognitive processes and is affected by numerous factors. Therefore, subsequent research can use the more accurate timing and spatial analysis provided by eye tracking technology [29] and ERP technology to explore the guiding mechanism of working memory attention. Second, Becker et al. [30] found that the number of colors in target-related stimuli of a search array had a large impact on the experimental results. In our two experiments, the color and shape of the target-related stimuli were all identical. Whether the current results can be obtained if the color or shape of the target-related stimuli in the search array is increased is worth further exploration. Finally, the simple geometry we used in the experiment may only access basic cognitive processing and may not be universally applicable to human attention in the complex real world. In subsequent research, experimental materials closely related to reality, such as natural scenes, real-life object images, or facial images [31] can be added to further explore the attention-based guidance mechanism based on working memory.

## 5. Conclusions

This study aimed to determine one way in which working memory can affect attention during searching and provide experimental evidence that could resolve disputes based on theories proposed in previous studies. By comparing search times for arrays containing different numbers of memory distractors as well as a baseline condition, Experiment 1 demonstrated that memory-matched stimuli may first capture the attention of the participant and then be suppressed by the participant. Experiment 2 excluded the possibility that increased search efficiency was due to the time needed to reject distractors. Based on the two experiments, we conclude that working memory content that matches non-target features will be processed in a search task and then suppressed.

## Figures and Tables

**Table 1:** Memory accuracies (%) by condition (M ± SDs).

Memory Task		
High	Low	Baseline
90.75 ± 5.48	92.48 ± 7.56	91.20 ± 4.80

**Table 2:** Search and memory task performance accuracy (%) (M ± SD).

	Border-Only Condition	Memory Condition	Memory and Border-Only Condition
Memory task	84.68 ± 10.23	84.29 ± 11.05	83.78 ± 12.19

## Data Availability

The datasets generated and/or analysed during the current study are not publicly available due to ongoing analysis for a follow-up study, but are available from the corresponding author on reasonable request.
